# Transcriptome Analysis of *Enterococcus faecalis* during Mammalian Infection Shows Cells Undergo Adaptation and Exist in a Stringent Response State

**DOI:** 10.1371/journal.pone.0115839

**Published:** 2014-12-29

**Authors:** Kristi L. Frank, Cristina Colomer-Winter, Suzanne M. Grindle, José A. Lemos, Patrick M. Schlievert, Gary M. Dunny

**Affiliations:** 1 Department of Microbiology, University of Minnesota Medical School, Minneapolis, Minnesota, United States of America; 2 Center for Oral Biology and Department of Microbiology and Immunology, University of Rochester School of Medicine and Dentistry, Rochester, New York, United States of America; University of Oklahoma Health Sciences Center, United States of America

## Abstract

As both a commensal and a major cause of healthcare-associated infections in humans, *Enterococcus faecalis* is a remarkably adaptable organism. We investigated how *E. faecalis* adapts in a mammalian host as a pathogen by characterizing changes in the transcriptome during infection in a rabbit model of subdermal abscess formation using transcriptional microarrays. The microarray experiments detected 222 and 291 differentially regulated genes in *E. faecalis* OG1RF at two and eight hours after subdermal chamber inoculation, respectively. The profile of significantly regulated genes at two hours post-inoculation included genes involved in stress response, metabolism, nutrient acquisition, and cell surface components, suggesting genome-wide adaptation to growth in an altered environment. At eight hours post-inoculation, 88% of the differentially expressed genes were down-regulated and matched a transcriptional profile consistent with a (p)ppGpp-mediated stringent response. Subsequent subdermal abscess infections with *E. faecalis* mutants lacking the (p)ppGpp synthetase/hydrolase RSH, the small synthetase RelQ, or both enzymes, suggest that intracellular (p)ppGpp levels, but not stringent response activation, influence persistence in the model. The ability of cells to synthesize (p)ppGpp was also found to be important for growth in human serum and whole blood. The data presented in this report provide the first genome-wide insights on *E. faecalis in* *vivo* gene expression and regulation measured by transcriptional profiling during infection in a mammalian host and show that (p)ppGpp levels affect viability of *E. faecalis* in multiple conditions relevant to mammalian infection. The subdermal abscess model can serve as a novel experimental system for studying the *E. faecalis* stringent response in the context of the mammalian immune system.

## Introduction

Many Gram-positive cocci, including staphylococci, streptococci, and enterococci, maintain commensal relationships with mammals by occupying specific biological niches, yet maintain the potential to become pathogenic when colonizing alternate sites within the same hosts. The enterococci, which naturally reside in the human gastrointestinal tract, are distinct from staphylococci and streptococci in the diverse range of non-mammalian environments that they inhabit, from soil and water to plants and the guts of insects [1]. Enterococci tolerate extreme temperatures and growth conditions that are generally unfavorable for other bacteria [1]. The inherent adaptability of enterococci is apparent in the breadth of physiologies these organisms achieve in order to thrive in such disparate environments.

*Enterococcus* spp. are the second most common pathogens associated with healthcare-associated infections in the United States, having caused more than 5500 central line-associated bloodstream infections, 3100 catheter-associated urinary tract infections, and 2400 surgical site infections in 2009–2010, as reported to the National Healthcare Safety Network [2]. *Enterococcus faecalis* is the species responsible for the highest percentage of enterococcal infections [2]. A number of virulence factors encoded on both the *E. faecalis* chromosome and on mobile genetic elements have been identified and characterized [3]. However, many studies report a lack of correlation between the presence of virulence factors in the genomes of isolates and the types of infectious or non-infectious sources from which the isolates originated [4]–[8]. These data suggest that a complex combination of factors contribute to the virulence of *E. faecalis*.

To date, transposon mutagenesis [9] and recombinase-based *in* *vivo* expression technology (RIVET) [10]–[12] screens are the only techniques that have been used for *in* *vivo* studies to identify *E. faecalis* genetic determinants involved in infection on a genome-wide scale. Transcriptomics approaches, such as microarray and RNA sequencing (RNA-seq), offer an alternative to genetic screens for evaluating the contributions of bacterial genes to *in* *vivo* growth at specific time points. Transcriptional profiling using microarrays has been performed for over a decade on numerous bacterial pathogens harvested directly from mammalian hosts [13]–[20], demonstrating that this methodology is widely applicable across bacterial species and animal models. RNA-seq has also been used to determine high resolution *in* *vivo* transcriptomes of pathogenic bacteria collected from animal infection models [21]–[23]. RNA-seq was recently used to compare the transcriptome of *E. faecalis* from the intestinal tracts of gnotobiotic mice to that of cells grown in BHI broth [24]. However, no *in* *vivo* transcriptomics studies of *E. faecalis* in a pathogenic setting have been conducted.

To gain a comprehensive view of the *E. faecalis* transcriptional landscape during the early stages of infection, we used microarrays to analyze changes in gene expression over time in a rabbit model of subdermal abscess formation. Substantial portions of the genome were differentially regulated at the sampled time points, with the resulting transcriptional profile at eight hours post-inoculation unexpectedly revealing hallmarks of a (p)ppGpp-mediated stringent response. The *in* *vivo* transcriptomics data resulting from this work were compared to the collection of *in* *vivo*-activated promoters identified in our previous subdermal abscess model RIVET screen [11] to gain further insight on *E. faecalis in* *vivo* gene expression and regulation. Together, these data provide a global description of the transcriptional profiles in *E. faecalis* that support physiological adaptation to growth in a mammalian host as a pathogen.

## Results and Discussion

### Survival of *E. faecalis* OG1RF in subdermal chambers during the course of infection

**Figure 1:**
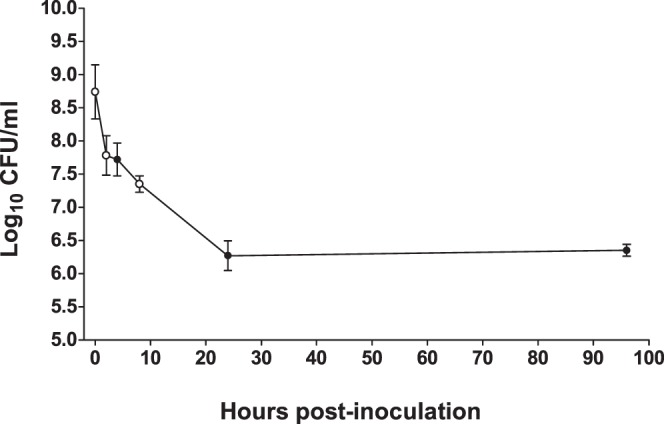
Recovery of *E. faecalis* OG1RF from subdermal abscesses during the course of infection. Two ml of *E. faecalis* OG1RF were inoculated into subdermal chambers from which two ml of serous fluid had been withdrawn. Dilutions of fluid aspirated from implanted chambers at the indicated time points following inoculation were plated to determine the number of viable *E. faecalis* CFU per ml recovered. The 0 h time points were calculated by dividing the total CFU in the two ml inocula (mean 1.65×10^10^ CFU, n = 4) by 30 ml, the approximate total volume of serous fluid in the implanted chamber. Results are reported as the log_10_ CFU/ml transformed values. Open circles indicate the time points analyzed by microarray (0, 2, and 8 hours). Symbols and error bars represent the mean ± SEM of n = 4 rabbits.

Our goal in this study was to gain insight on the process of *E. faecalis* adaptation to a host environment as a pathogen during the early stages of infection. We previously used the rabbit subdermal abscess model for a RIVET genetic screen to identify promoters in *E. faecalis* that are specifically up-regulated in infection [11]. Since the model enables sampling of the same infection site over time and has been used for *Staphylococcus aureus in* *vivo* gene expression profiling [25], we used the model in this work to continue our investigations of *E. faecalis* OG1RF gene expression during infection. Chambers were inoculated to contain an average of 8.7 log_10_ CFU/ml OG1RF and were followed for 96 hours (Fig. 1). The number of OG1RF cells recovered from chambers showed an initial decrease of one log_10_ CFU/ml over the first two to four hours, followed by an additional drop of 0.4 log_10_ CFU/ml between four and eight hours. Cell recovery further declined to 6.3 log_10_ CFU/ml at 24 hours and remained at that level through the final sample collection at 96 hours post-inoculation. The 2.4 log_10_ CFU/ml decrease over the first 24 hours of infection suggests that bacteria were eliminated from the chamber by the local immune cell population, which was historically reported to be comprised of ∼10% polymorphonuclear cells and 90% mononuclear cells [26].

### Use of microarray to detect differential gene expression in *E. faecalis* OG1RF during subdermal abscess infection

The RIVET screens that we and others previously carried out in rabbit subdermal chamber abscesses [11], murine bacteremia and peritonitis [10], the *Manduca sexta* gut [12], and *G. mellonella* larvae hemocoel [10] proved useful for identifying specific *E. faecalis in* *vivo*-activated promoters. However, these screens are limited by the low number of overlapping genes identified among different hosts [11], minimal data on the specific temporal expression patterns of *in* *vivo*-activated genes, and the inability to identify genes that are negatively regulated *in* *vivo*. As a result, we anticipated that genome-wide gene expression analyses would provide a novel perspective on the transcriptional changes that take place in *E. faecalis* in the transition from *in* *vitro* to *in* *vivo* pathogenic growth. Since OG1RF cells were challenged by the host immune response during the early hours of infection in the subdermal chambers, we decided to compare gene expression in the input inoculum with that of cells recovered at two and eight hours post-inoculation by microarray (Fig. 1, open circles).

Analysis of transcripts collected two hours post-inoculation revealed that 222 genes, representing 9.5% of the total OG1RF open reading frames (ORFs) assayed on the array, were differentially regulated by two-fold or greater (p-value <0.05) when compared to the initial inoculum (S1 Table). Of the 222 genes, 117 genes (53%) were up-regulated and 105 genes (47%) were down-regulated. At eight hours post-inoculation, 291 genes (12.4% of all analyzed OG1RF ORFs) were differentially regulated (S2 Table). Eighty-eight percent (n = 257) of these genes were down-regulated, while only 12% (n = 34) of genes were up-regulated. Only three up-regulated genes and 31 down-regulated genes were in common between the two and eight hour data sets (column H in S1 and S2 Tables), suggesting that the transcriptional profile in *E. faecalis* cells underwent a major shift between the two time points.

To validate the microarray results, quantitative PCR (qPCR) for 13 differentially regulated genes was performed on reverse-transcribed RNA collected from subdermal chambers at two and eight hours post-infection (S1 Fig.). Expression data for 12/13 genes at two hours and 11/13 genes at eight hours were concordant between the two techniques.

**Figure 2:**
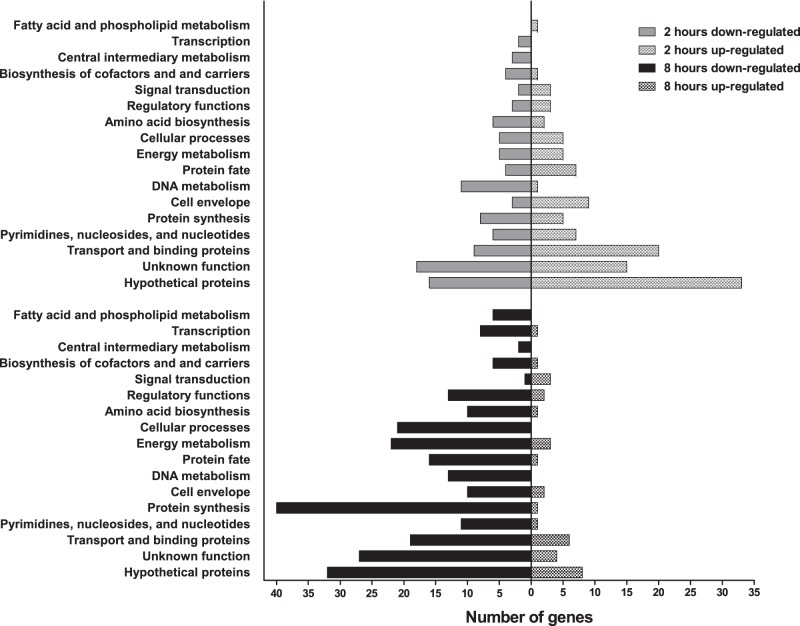
Functional categories of genes found by microarray to be differentially expressed in *E. faecalis* OG1RF *in* *vivo* during subdermal abscess infection. The numbers of up- (right) and down-regulated (left) genes at two (top) or eight (bottom) hours post-inoculation are indicated on the horizontal axis.

The significant differentially regulated genes at each time point are graphed in Fig. 2 according to their functional categories (S1 and S2 Tables), which were assigned following the scheme used by the J. Craig Venter Institute [27], [28] for the *E. faecalis* V583 genome [29]. Notably, 37% of the genes at two hours, and 24% of the genes at eight hours, encode proteins with annotations of hypothetical or unknown functions. The functional categories with the next-highest number of up- and down-regulated genes at two hours (Fig. 2A) were transport and binding proteins (n = 20) and DNA metabolism proteins (n = 11), respectively. The down-regulated genes at eight hours were distributed across all of the functional categories (Fig. 2B), with the highest number of genes occurring in the protein synthesis category (n = 40).

### *E. faecalis* OG1RF adaptation during the initial hours of growth in a mammalian host is the result of genome-wide transcriptional changes

The differentially regulated genes in the subdermal chambers at two hours revealed genome-wide transcriptional changes suggestive of cellular adaptation to growth in an altered environment. Groups of genes involved in stress response, metabolism, nutrient acquisition, and cell surface components were differentially expressed (S1 Table). Six *E. faecalis* stress response genes that were up-regulated at two hours in the subdermal chambers were similarly up-regulated upon *in* *vitro* exposure to defibrinated horse blood [30]. These genes included *gls24* and *glsB* (EF0079–EF0080), the first of which contributes to virulence [31], [32] and both of which impact cellular stress response [33]; two universal stress proteins (EF1058 and EF1084); a hypothetical gene (EF1560); and *clpB* (EF2355), which confers thermotolerance and virulence in a *Galleria mellonella* infection model [34]. The genes encoding chaperone GroEL and chaperonin GroES (EF2633-2634) were also coordinately up-regulated in the rabbit, while the gene for the cold shock protein CspA (EF1367) was down-regulated.

Multiple operons contributing to metabolic processes were down-regulated at two hours. Five genes in the co-transcribed region spanning EF1561 to EF1568, which encode the enzymes of the shikimate pathway in *E. faecalis*, were among the most strongly down-regulated genes in the two hour microarray data set. Microbes and plants use the shikimate pathway to synthesize aromatic amino acids and aromatic secondary metabolites from carbohydrates through the precursor molecule chorismate [35]. Two genes in the EF0445-0450 operon dedicated to the synthesis of menaquinone from chorismate were correspondingly down-regulated at two hours. Vebø et al. also reported down-regulation of the EF1561-1568 and EF0445-0450 regions in *E. faecalis* V583 following incubation in horse blood [30]. Multiple arginine catabolism genes (EF0104, EF0106-0107) were also down-regulated at two hours (S1 Table).

Large numbers of genes in the transport and binding proteins functional category were up-regulated genes at two hours post-infection (Fig. 2A). This is consistent with a bacterial response to changing nutrient availabilities in the environment. The most highly up-regulated group of genes at two hours was EF1219 to EF1223 (S1 Table), which encodes components of a spermidine/putrescine transport system (EF1219-EF1221), adenine deaminase (EF1222), and a putative S-adenosylhomocysteine deaminase (EF1223). Adenine deaminase and polyamine transport systems have been shown to be active in other Gram positive species when extracellular adenine and polyamines, respectively, were abundant [36], [37]. The genes for four iron transport proteins were also up-regulated (EF0475-0476, EF3082, and EF3085). In particular, transcription of EF3082, a ferric ABC transporter binding protein, and EF3085, an ABC transporter membrane permease, was increased by 23.5- and 42.7-fold, respectively. Transcription of both genes was also significantly increased in cells grown in horse blood [30] and in broth under iron-limiting conditions [38].

*E. faecalis* produces a plethora of surface proteins [39], [40], including dozens of lipoproteins [41], that likely mediate interactions with the host. The surface proteins Ebp pili and Ace, a collagen binding protein, are important enterococcal virulence factors [42], but transcription was not significantly changed for either gene in the subdermal chambers at two hours. Nine lipoproteins were up-regulated at two hours. The differentially regulated lipoproteins belonged to the transport and binding proteins (EF0063, EF0176, EF0177, EF1221, EF2076, EF3082), cell envelope (EF0095), hypothetical proteins (EF1085), and protein fate (EF0685) functional categories (S1 Table). Several genes in the *dlt* operon [43], [44]–*dltX* (EF2750), *dltA* (EF2749), and *dltC* (EF2747)–which incorporates D-alanine into lipoteichoic acid and wall teichoic acid, were up-regulated at two hours post-inoculation. The gene for alanine racemase (EF0849), the enzyme that generates D-alanine from L-alanine and is integral for cell wall synthesis [45], was also up-regulated at two hours.

Our use of the subdermal abscess infection model in this work provided the benefit of studying *E. faecalis* transcription in response to the host innate immune system in the presence of a foreign body. However, a limitation of the model is that the results may not be predictive of *E. faecalis* gene expression patterns in other types of infection models, such as bacteremia or urinary tract infection, due to differences in nutrient availability in the respective environments (e.g., blood and urine). *In vivo* gene expression studies in such models would be of interest, as *E. faecalis* is a common cause of bloodstream and catheter-associated urinary tract infections [2]. Nevertheless, the early time-point *in* *vivo* transcriptional profiles presented here significantly expand on previous studies characterizing *E. faecalis* gene expression following *in* *vitro* incubation in biological fluids [30], [46], [47]. In the experiment that most closely resembled the conditions we tested, 549 genes in the vancomycin-resistant *E. faecalis* strain V583 were differentially expressed after incubation in 100% defibrinated horse blood for 30 minutes [30]. The adaptive response of V583 to blood includes transcriptional changes in genes coding for stress protection, cell envelope maintenance, metabolism, transport and binding, and amino acid biosynthesis proteins. Despite the lack of the host immune response *in* *vitro*, the difference in time points and strains studied, and bacterial exposure to blood *in* *vitro* and serous fluid *in* *vivo*, our two hour microarray data showed similar transcriptional responses (S1 Table and Fig. 2). The extensive involvement of genes that code for metabolism, biosynthesis, and transport and binding proteins in either experiment is indicative of bacterial physiological shifts in response to nutrient availability in the respective growth environments. Notably, proteins with functions in glycolysis/gluconeogenesis, virulence, stress, and iron acquisition were detected by proteomic analysis of *S. aureus* abscesses in neutropenic mice [48], suggesting that many of the same responses occur among Gram-positive pathogens during abscess infections.

### Identification of a stringent response transcriptional profile during subdermal abscess infection

By eight hours post-infection, at which point the bacterial cell numbers had dropped below the inoculum by 1.4 log_10_ CFU/ml (Fig. 1), we observed down-regulated transcription of many components of fundamental cellular pathways required for growth and replication (S2 Table). The down-regulated genes included: 23 ribosomal protein genes, eight aminoacyl tRNA ligase genes, five subunits of the ATP synthase F_0_ and F_1_ machinery, DNA polymerase III (δ’ subunit), DNA gyrase (B subunit), DNA topoisomerase (A subunit), replicative DNA helicase DnaB, DNA primase, the δ and Ω subunits of RNA polymerase, and sigma factor RpoD. Global shut-down of such processes is consistent with the stringent response elicited among bacteria during adverse conditions [49]. Indeed, over 21% (62/291) of the *E. faecalis* eight hour differentially regulated genes were identical to, or were the same types of genes as (e.g., ribosomal protein genes), the transcriptional profile of the *E. coli* stringent response induced after amino acid starvation (marked in blue font in S2 Table) [50]. The stringent response is governed by the accumulation of the metabolite (p)ppGpp during periods of stress that are unsuitable for growth, including nutrient deprivation [49]. One hundred twenty-three genes in *E. faecalis* OG1RF were found to be under (p)ppGpp-positive control during stringent response activation following 15 or 30 minute treatment with mupirocin [51]. Fifty-seven of the down-regulated genes and two of the up-regulated genes in the eight hour microarray (20.3%) overlapped this set of *in* *vitro* (p)ppGpp-controlled genes (marked in boldface text in S2 Table). From these data, we conclude that the population of OG1RF cells in the subdermal chambers at eight hours after inoculation is in a stringent response state. The low overlap of up-regulated genes between the mupirocin-induced and *in* *vivo* stringent response transcriptional profiles may be due, in part, to genes that were discarded during data analysis (see Materials and Methods). More so, the strong and rapid stringent response induction achieved under artificial conditions *in* *vitro* with mupirocin may have resulted in a greater number of differentially regulated genes overall. This has been observed in *Escherichia coli*, where gene expression patterns change directly in response to intracellular concentrations of ppGpp following stringent response induction [52].

It is important to note that the time at which the stringent response was activated cannot be discerned from these microarray data. An unknown source of stress *in* *vivo*, such as selective nutrient deprivation or one or more components of the host innate immune response (e.g., oxidative stress), may have contributed to *in* *vivo* activation of the stringent response in *E. faecalis* cells between two and eight hours post-inoculation. Alternatively, a minority of cells in the population may have entered a stringent response state much earlier. A situation like this has been reported to happen in *E. coli*, where persister cells form as a result of stochastic variation in (p)ppGpp levels [53]. In this scenario, a stringent response-induced subpopulation of *E. faecalis* in the subdermal abscesses would have contributed to the total gene expression measured in the two hour microarray, yet the stringent response signal would have been masked by the RNA patterns from the majority of the cells in the population, which revealed cellular adaptation to the altered environment. The transcriptome of stringent response-induced cells of such a subpopulation may have become measurable at eight hours due to the overall reduction of viable bacteria (Fig. 1) over the same period. However, the difference in viable bacteria between two and eight hours was only ∼0.5 log_10_ CFU/ml. Therefore, further experimentation will be required to test this hypothesis.

In *E. faecalis*, (p)ppGpp levels are maintained by two enzymes, the bifunctional synthetase/hydrolase RSH (also called RelA) and the small synthetase RelQ, which are encoded by genes EF1974 and EF2671, respectively [54], [55]. RSH produces (p)ppGpp during stress conditions, while RelQ maintains (p)ppGpp pools during homeostatic growth [55]. Neither gene was statistically differentially expressed in the microarray analyses (S1 and S2 Tables), which is consistent with the lack of detection of either gene in microarray experiments carried out previously on mupirocin-treated cells [51]. Using reverse transcription-qPCR, we found that transcript levels of *rsh* were unchanged (fold change 1.2±1.3 standard deviation, n = 5) and *relQ* were slightly increased (fold change 2.0±0.8 standard deviation, n = 5) at eight hours post-infection versus the inoculum. The *relQ* promoter was also found to be up-regulated in the subdermal abscess model in our previous RIVET screen [11], providing additional evidence that the locus is subject to transcriptional regulation under *in* *vivo* conditions. Geiger et al. recently showed that transcription of the *S. aureus relQ* locus, along with a second short (p)ppGpp synthase gene, *relP*, is strongly activated by the cell wall-targeting antibiotics vancomycin and ampicillin [56]. Similarly, the *Bacillus subtilis* (p)ppGpp synthase gene *relQ* (also known as *ywaC*) is transcriptionally up-regulated in response to alkaline shock [57]. These observations are consistent with the notion that the enzymes involved in (p)ppGpp metabolism are mostly under allosteric regulation.

### Contribution of *E. faecalis* rsh and relQ to survival under *in vivo* and *ex vivo* conditions

During *in* *vitro* growth, deletion of *rsh* increases basal levels of ppGpp and simultaneously decreases GTP pools [58]. This is associated with a slow growth phenotype in unstressed conditions [55] and significant growth delays in 5% sodium chloride, acidic pH, and 2 mM hydrogen peroxide [55]. In contrast, deletion of *relQ* in OG1RF does not appear to affect basal pools of (p)ppGpp or GTP [58], but causes delayed onset of stringent response-related transcriptional changes [51] and increases susceptibility to vancomycin [55]. No virulence attenuation phenotypes were found when OG1RF Δ*rsh* and Δ*relQ* deletion strains were tested in *Caenorhabditis elegans* [55] or *G. mellonella* [51]. The OG1RF (p)ppGpp^0^ strain (Δ*rsh*Δ*relQ*), however, is attenuated for virulence in both invertebrate models, survival in macrophages, and killing by vancomycin, norfloxacin, and ampicillin [51], [55], [58].

**Figure 3:**
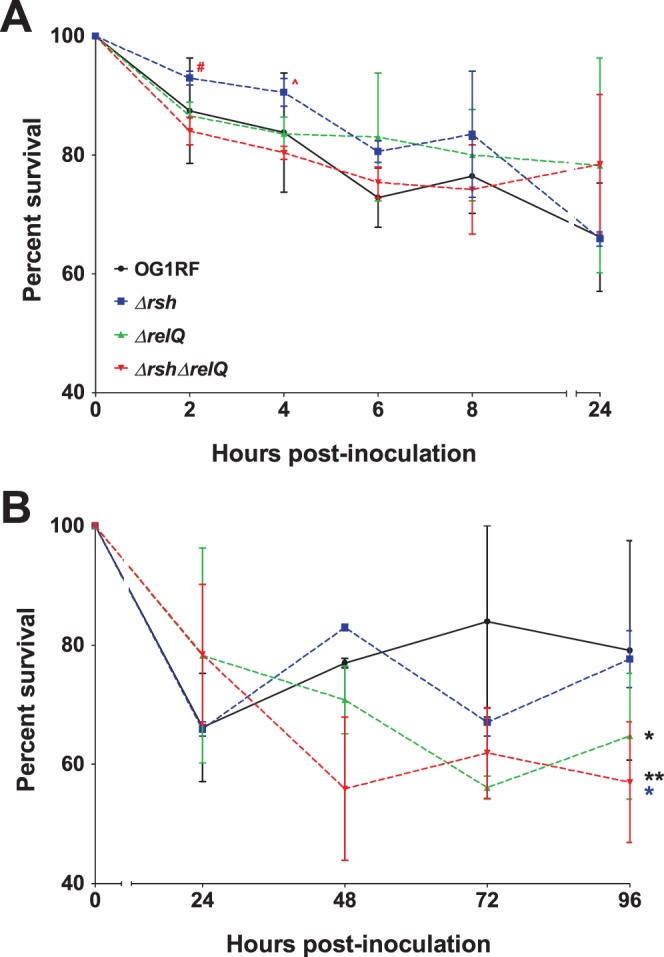
Survival of *E. faecalis* OG1RF, Δ*rsh*, Δ*relQ*, and Δ*rsh*Δ*relQ* in subdermal abscesses at (A) early and (B) late time points post-inoculation. Subdermal abscess infections with the four strains were carried out as described in the text and in the legend of Fig. 1. Results are reported as percent survival with the 0 hour time point set to 100%. Values and error bars represent the mean ± SEM of n = 2 rabbits. Data from the same rabbits were separated into panels (A) and (B) for clarity. (A) Student’s t-test comparing Δ*rsh* and Δ*rsh*Δ*relQ*, α = 0.1: red #, p = 0.08; red ∧, p = 0.06. (B) One-way ANOVA followed by Tukey’s Multiple Comparison post-hoc test: black *, p<0.05 for OG1RF versus Δ*relQ*; black **, p<0.01 for OG1RF versus Δ*rsh*Δ*relQ*; blue *, p<0.05 for Δ*rsh* versus Δ*rsh*Δ*relQ*.

The contributions of the *E. faecalis rsh* and *relQ* genes to mammalian infection have not been studied previously. We used OG1RF Δ*rsh*, Δ*relQ*, Δ*rsh*Δ*relQ* in-frame deletion mutants to evaluate the roles of RSH and RelQ, and thereby production of (p)ppGpp, in survival in the subdermal chambers. The percent survival of each strain, which was measured in the chambers during the initial hours of infection and then daily for four days post-infection, is shown in Fig. 3. The cell recovery in log_10_ CFU/ml is shown in S2 Fig. Since both the Δ*rsh* and Δ*rsh*Δ*relQ* strains are unable to mount a stringent response [55], these experimental infections allowed us to test the hypothesis that stringent response activation is necessary for the persistence phenotype observed for OG1RF in the subdermal chambers at 96 hours post-infection (Fig. 1).

As observed with OG1RF (Figs. 1 and 3), recovery of the three in-frame deletion mutants decreased during early infection (Fig. 3A) and then persisted in the subdermal chambers for the duration of the infection (Fig. 3B). None of the strains, regardless of their ability to mount a stringent response, were cleared from the chambers. However, the (p)ppGpp mutant strains showed disparate phenotypes at the early (Fig. 3A) and prolonged (Fig. 3B) stages of infection. The rate of clearance of the Δ*rsh* strain was diminished at two and four hours compared to the other strains. These differences were particularly pronounced with the Δ*rsh*Δ*relQ* strain (Fig. 3A, α = 0.1, p = 0.08 and 0.06 at two and four hours, respectively). Between 48 to 96 hours, the four strains persisted at relatively stable levels that fluctuated daily (Fig. 3B). During that period, OG1RF persisted at a mean percent survival of 80.0±15.5% (standard deviation). The mean percent survival of Δ*rsh* was nearly identical to OG1RF (75.9±8.0%), while the mean percent survival levels of Δ*relQ* and Δ*rsh*Δ*relQ* (63.9±10.1% and 58.3±11.4%, respectively) were significantly lower than OG1RF (p<0.05 for Δ*relQ* and p<0.01 for Δ*rsh*Δ*relQ*). The difference between the Δ*rsh* and Δ*rsh*Δ*relQ* strains was also significant (p<0.05).

**Figure 4:**
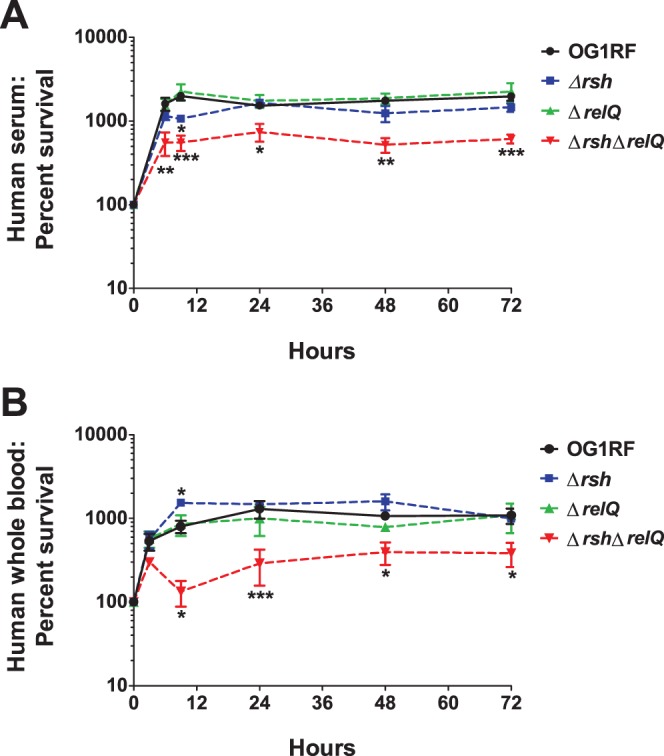
Growth and survival of *E. faecalis* OG1RF, Δ*rsh*, Δ*relQ*, and Δ*rsh*Δ*relQ* in human (A) serum and (B) whole blood *ex vivo*. Overnight cultures of the four strains were diluted 1∶20 in serum or whole blood and incubated at 37°C. Dilutions of aliquots collected at 0, 3 (panel B), 6 (panel A), 9, 24, 48, and 72 hours were plated to assess bacterial survival. Results are reported as percent survival with the 0 hour time point set to 100%. Values and error bars represent the mean ± SEM of n = 3 independent replicates. Data were analyzed for statistical significance with two-way ANOVA followed by Dunnett’s multiple comparisons test. *, p<0.05; **, p<0.01; ***, p<0.001.

We also compared growth and survival of OG1RF and the three (p)ppGpp-defective strains in human serum (Fig. 4A) and whole blood (Fig. 4B) *ex vivo* (Fig. 4). The growth kinetics and prolonged survival levels of OG1RF and the Δ*relQ* strain were similar in serum and blood. In contrast, growth of the Δ*rsh* strain at nine hours was significantly lower than growth of OG1RF in serum (Fig. 4A), but was significantly higher than growth of OG1RF in blood (Fig. 4B). By 24 hours, survival levels of the Δ*rsh* strain were essentially indistinguishable from OG1RF and the Δ*relQ* strain in either condition. Growth of the Δ*rsh*Δ*relQ* strain was significantly lower than OG1RF at all nearly all time points studied in serum and blood.

Taken together, these data show that intracellular (p)ppGpp levels affect viability of *E. faecalis* in multiple conditions relevant to mammalian infection. The ability of a strain to activate a stringent response does not influence persistence in this model, as evidenced by the fact that two strains that can mount a stringent response (OG1RF wild type and Δ*relQ*) and two strains that cannot mount a stringent response (Δ*rsh* and Δ*rsh*Δ*relQ*) persisted in the subdermal chambers for the 96 hour infection. Gaca et al. previously reported impaired survival of the (p)ppGpp^0^ strain (Δ*rsh*Δ*relQ*) in macrophages [51]. Consistent with this, our data confirm that the complete lack of (p)ppGpp impairs growth and survival in subdermal abscesses, serum, and blood. The different survival phenotypes for the Δ*relQ* strain in the subdermal abscess model (Fig. 3B) and the *ex vivo* serum and blood experiments (Fig. 4) suggest the hypothesis that RelQ may contribute to host-pathogen interactions that were not intact in the *ex vivo* assays. Finally, the decreased clearance and increased growth rates of the Δ*rsh* strain in subdermal abscesses (Fig. 3A) and in whole blood (Fig. 4B), respectively, support a model in which high basal levels of ppGpp, and correspondingly low levels of GTP, confer a protective advantage from the host innate immunity. Follow-up studies will be essential to decipher the complete role of (p)ppGpp in the pathogenesis of *E. faecalis* infections.

### Insights on *E. faecalis in vivo* gene expression gained from complementary techniques

**Figure 5:**
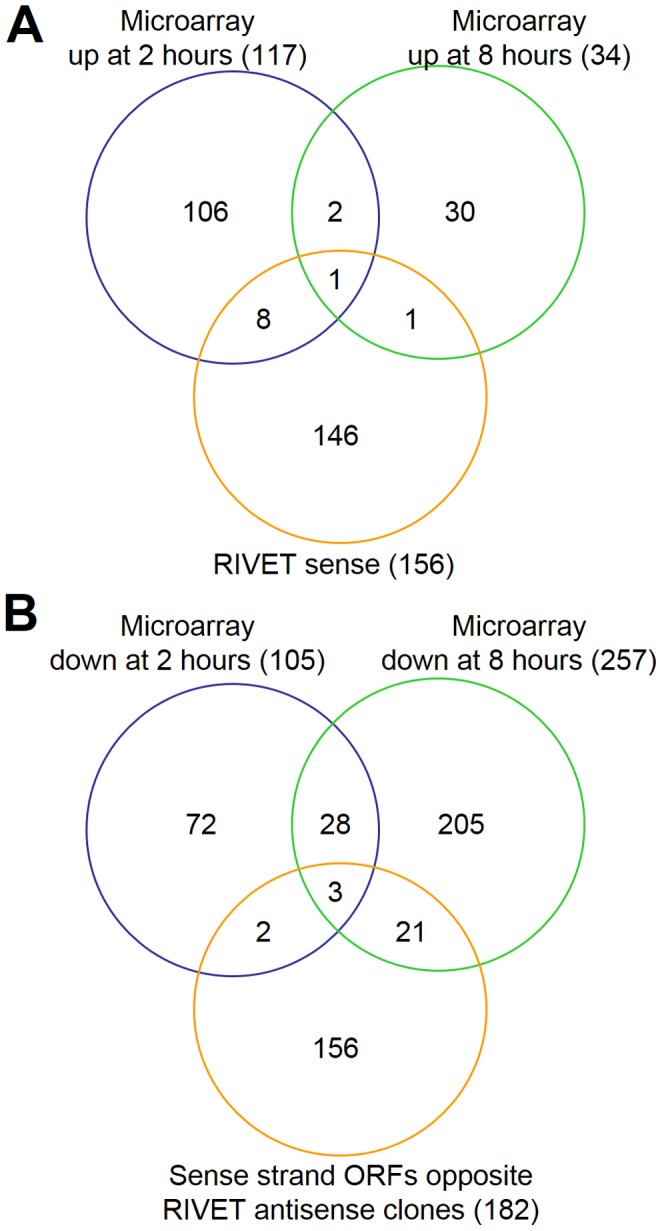
Overlap of differential gene expression between microarray and *in* *vivo*-activated transcripts identified by RIVET screen in the *E. faecalis* subdermal abscess model [11]. (A) Up-regulated genes identified by microarray compared with RIVET sense direction clones. (B) Down-regulated genes identified by microarray compared with sense strand ORFs that are complementary to RIVET antisense clones. See text and reference [11] for additional details on the subdermal abscess RIVET screen that was previously conducted. See Table 1 for the identities of the genes from microarray analyses that overlap with RIVET analysis genes. See column H of S1 and S2 Tables for the identities of genes found in both microarray analyses.

To gain additional insight on *E. faecalis* gene regulation in the subdermal abscess environment, we compared the microarray data from this study with our previous RIVET screen data set [11]. That screen identified 249 *in* *vivo*-activated promoters in *E. faecalis* OG1RF during the early hours of infection in the subdermal abscess model. One hundred fifty-six *in* *vivo*-activated promoters are predicted to generate transcripts from the sense strand, with the remainder of the promoters driving expression from the antisense strand. The subdermal abscess RIVET sense strand gene set was compared to the up-regulated genes from the microarray analysis at two and eight hours post-inoculation (Fig. 5A and Table 1). Ten genes were shared between the RIVET screen and either microarray condition, with a single gene, EF1672 (permease protein), activated in all three conditions tested. Interestingly, EF1672 was also one of the two up-regulated genes in the eight hour microarray that overlapped the set of genes regulated in a (p)ppGpp-dependent manner during *in* *vitro* stringent response activation (marked in boldface text in S2 Table) [51]. Overall, only a fraction of the RIVET clones overlapped with the differentially expressed genes (Fig. 5). Differences in the time points sampled (i.e., two and eight hours post-inoculation for microarray versus four, eight, and 24 hours post-inoculation for RIVET) and in the microarray and RIVET methodologies (e.g., RIVET clones may represent promoters that are expressed transiently or in only a minor subpopulation of cells) may account, in part, for the broad coverage of differentially regulated genes that comprise the two data sets. Regardless, the complementary and largely non-redundant outcome of pairing the data sets permitted the discrete identification of candidate genes that we hypothesize represent core genetic determinants necessary for *E. faecalis* adaptation to growth in a host (Fig. 5A and Table 1). Future functional studies of EF1672 and the other candidate core genes in Table 1 may lead to the identification of new targets for antimicrobial agents or vaccines for the treatment and prevention of enterococcal infections.

**Table 1:** Differentially regulated genes in subdermal abscesses identified by microarray that overlap with a previously conducted subdermal abscess RIVET screen [11].

Category^1^	Locus	Gene Description
2 h microarray UP +8 h microarray UP+RIVET sense		
	EF1672	permease protein
2 h microarray UP+RIVET sense		
	EF0031	hypothetical protein
	EF0246	ABC superfamily ATP binding cassette transporter, ABC protein
	EF0475	ferrous iron transport protein A
	EF0697	hypothetical protein
	EF0849	alanine racemase
	EF1084	universal stress protein
	EF2355	chaperone protein ClpB
	EF2987	integrating conjugative element protein
8 h microarray UP+RIVET sense		
	EF0383	hypothetical protein
2 h microarray DOWN +8 hmicroarray DOWN+RIVET antisense		
	EF1360	dihydroxyacetone kinase
	EF1614	DNA topoisomerase subunit A
	EF2623	P-ATPase superfamily P-type ATPase cadmium transporter
2 h microarray DOWN+RIVET antisense		
	EF0004	recombination protein F
	EF1112	exonuclease RexB
8 h microarray DOWN+RIVET antisense		
	EF0005	DNA gyrase subunit B
	EF0013	replicative DNA helicase DnaB
	EF0225	50S ribosomal protein L30
	EF0226	50S ribosomal protein L15
	EF0724	glutamyl-tRNA(Gln) amidotransferase subunit C
	EF0801	leucyl-tRNA synthetase
	EF0909	oligopeptide ABC superfamily ATP binding cassette transporter, membrane protein
	EF0988	cell division protein MraZ
	EF1003	isoleucine–tRNA ligase
	EF1146	DNA-directed RNA polymerase sigma subunit RpoE
	EF1361	dihydroxyacetone kinase
	EF1522	RNA polymerase sigma factor rpoD
	EF1550	DNA-binding protein HU
	EF1580	hypothetical protein
	EF1629	ethanolamine ammonia-lyase large subunit
	EF2397	elongation factor EF1B
	EF2398	30S ribosomal protein S2
	EF2908	group 2 glycosyl transferase
	EF3178	succinyl-diaminopimelate desuccinylase
	EF3270	glutathione-disulfide reductase
	EF3278	hypothetical protein

1 Microarray UP and Microarray DOWN indicate that the locus was up- or down-regulated, respectively, in microarray analysis at the indicated time point.

The high number of *in* *vivo*-activated antisense promoter regions (n = 93) identified in the subdermal abscess RIVET screen [11] led us to hypothesize that *E. faecalis* may use global antisense gene regulation mechanisms during *in* *vivo* growth. We reasoned that if antisense transcripts predicted by the RIVET clones were acting to silence transcription, there might be a correlation between the down-regulated transcripts identified by microarray and the subdermal abscess RIVET antisense transcript list. Many of the 93 predicted antisense promoter regions from the RIVET screen overlap more than one sense strand ORF, resulting in a total of 182 sense strand ORFs potentially affected by an antisense transcript (see reference [11]). Therefore, we compared the down-regulated microarray gene lists to this set of 182 ORFs (Fig. 5B and Table 1). Twenty-six genes overlapped in the gene sets, with three genes (Table 1) shared in all three conditions. The majority of the remaining overlapping genes were from the eight hour microarray, ten of which were stringent response-related genes (Fig. 5B and Table 1). Two of these genes, EF0909 and EF2398, are complementary to confirmed antisense transcripts that are expressed *in* *vivo* during subdermal abscess infection [11]. In total, 4.8% of the down-regulated genes at two hours and 9.3% of down-regulated genes at eight hours corresponded with antisense clones identified in the subdermal abscess RIVET screen. Further studies are needed to determine whether these predicted sense-antisense interactions occur within *E. faecalis* and, if so, whether they are targets for RNase III cleavage, which has been proposed as a post-transcriptional regulatory mechanism in Gram-positive bacteria [59].

## Conclusions

In conclusion, this report provides a comprehensive characterization of the gene expression changes in *E. faecalis* during the course of a subdermal abscess infection in a mammalian host. This is the first description of the use of microarrays to evaluate *E. faecalis in* *vivo* transcription in a vertebrate infection model (S1 and S2 Tables). Our analysis reveals that *in* *vivo*-grown *E. faecalis* cells existed in two distinct transcriptional states during the first several hours of infection (Figs. 2 and 4, and S1 and S2 Tables): the enterococci first adjusted to growth in a new environment followed by the surviving cells reaching a stringent response state. Our genetic data suggest that this physiologic state is important for survival in this environment and that it may have resulted from high levels of (p)ppGpp. In addition, the combined RIVET and microarray data analysis is in support of our hypothesis that *E. faecalis* may use antisense transcripts to globally silence expression of sense strand transcripts *in* *vivo*. Finally, our serendipitous observation of an *E. faecalis in* *vivo* stringent response transcriptome suggests that the subdermal chamber infection model can serve as a novel experimental system for studying the role of (p)ppGpp in the context of the mammalian immune system.

## Materials and Methods

### Bacterial strains, growth conditions, and enzymes

The *E. faecalis* OG1RF strain background [58], [60] was used for all experiments. OG1RF Δ*rsh* (formerly called Δ*relA* [58]), OG1RF Δ*relQ*, and OG1RF Δ*rsh*Δ*relQ* were described previously [55]. Bacteria were streaked onto Brain Heart Infusion (BHI, BD Bacto, Becton, Dickinson and Company, Sparks, MD) agar plates from glycerol stocks stored at -80°C and incubated at 37°C. Cultures for animal experiments were grown in trypsinized beef heart dialysate (BH) medium [61] under static conditions at 37°C. Lysozyme and mutanolysin were purchased from Sigma-Aldrich (St. Louis, MO).

### Rabbit model of subdermal abscess formation

All animal procedures were carried out in accordance with the guidelines set forth by the Public Health Service Policy on Humane Care and Use of Laboratory Animals. The University of Minnesota Institutional Animal Care and Use Committee approved the protocol used in this work (protocol 0910A73332). Animals were euthanized with Beuthanasia-D and efforts were made to minimize suffering.

Subdermal chambers were implanted subcutaneously into the flanks of New Zealand white rabbits (2–3 kg, either sex) exactly as previously described [11]. The recovery period lasted a minimum of six weeks in order for the implanted chambers to be encapsulated with fibrous tissue and fill with approximately 30 ml of serous fluid. Microarray analysis samples were obtained from four rabbits in which chambers had been implanted 7 to 45 weeks prior to infection. The (p)ppGpp mutant strains were studied in two rabbits per strain in which chambers had been implanted 7 weeks prior to infection. To initiate infection, two ml of serous fluid were aspirated from the subdermal chamber and were replaced with 2 ml of inoculum prepared as described below for gene expression analyses. Bacterial recovery, measured as CFU/ml, was assessed as described below. Results are reported as the log_10_ CFU/ml transformed values or as percent survival with the bacterial load at the 0 hour time point set to 100%.

### Subdermal chamber infections for survival counts and gene expression analyses

*(A) Microarray experiments.* A single colony of *E. faecalis* OG1RF was inoculated into 10–25 ml BH medium and incubated for ∼15–16 hours, then diluted 1∶5 into 25–50 ml of fresh BH medium and incubated for 2 more hours. Bacteria were harvested by centrifugation for 15–20 min at 6000 rpm in a Beckman JA-17 rotor at 4°C. Pelleted cells were resuspended to an optical density at a wavelength of 600 nm of ∼1.3–1.6 in KPBS, and 2 ml were used for each subdermal chamber infection. Two milliliter volumes were removed from the subdermal chambers at 2, 4, 8, 24, and 96 hours post-inoculation. Approximately 0.1–0.2 ml aliquots of the initial inoculum and each aspirate were used for serial dilution and plating to quantitate the bacterial load at each time point. Two milliliters of the initial inoculum, and the remainder of each aspirate (∼1.8 ml), were immediately added to 4 ml of RNAprotect Bacteria Reagent (Qiagen Inc., Valencia, CA), vortexed, processed according to the manufacturer’s instructions, flash-frozen in a dry ice/ethanol bath, and stored at −80°C until RNA extraction. *(B) Infections with (p)ppGpp mutant strains.* Infections with OG1RF and the OG1RF deletion strains Δ*rsh*, Δ*relQ*, and Δ*rsh*Δ*relQ* were carried out as described above for microarray experiments, except that aspirates were collected from the subdermal chambers at 2, 4, 6, 8, 24, 48, 72, and 96 hours post-inoculation. Statistical comparisons were calculated with the Student’s t-test function in Microsoft Excel 2013 and one-way ANOVA followed by Tukey’s Multiple Comparison post-hoc test in Graphpad Prism (version 5.04). Significance was assessed at α = 0.05 unless otherwise stated.

### RNA extraction and DNase treatment

RNA was prepared from subdermal aspirates as follows: frozen pellets were thawed, resuspended in 0.4 ml of RNase-free TE containing 50 mg/ml lysozyme and 1000 U/ml mutanolysin, homogenized using a hand-held motorized homogenizer with RNase-free pellet pestles (Fisher Scientific, Pittsburgh, PA), and incubated for 10 min at 37°C. Each sample was then split in half and extracted in duplicate with the RNeasy Mini Kit (Qiagen Inc.) according to the manufacturer’s instructions with an added homogenization step using the QIAshredder (Qiagen Inc.) immediately after the addition of Buffer RLT. RNA was eluted from each column with two 30 µl volumes of RNase-free water, and total RNA from duplicate extractions of each sample were pooled together. RNA from uninfected serous fluid was extracted with TRIzol Reagent (Invitrogen Corp., Carlsbad, CA) as suggested by the manufacturer immediately following aspiration from the subdermal chamber.

Contaminating DNA was removed using a TURBO DNA-free kit (Ambion, Austin, TX) following the rigorous protocol as directed by the manufacturer. RNA for microarray experiments was then ethanol precipitated in the presence of a glycogen carrier (Roche Applied Science, Indianapolis, IN) and resuspended in 10 µl RNase-free water; 1 µl was reserved for qPCR experiments.

### Genomic DNA (gDNA) extraction and shearing

gDNA from a 50 ml culture of *E. faecalis* OG1RF grown overnight in Todd-Hewitt broth (BD Bacto, Becton, Dickinson and Company) was extracted using a Genomic-tip 500/G column (Qiagen, Inc.) as directed by the manufacturer. Precipitated DNA was resuspended in 1.7 ml of 10 mM Tris-Cl, pH 8.5. Approximately 425 µg of gDNA were sheared by nebulization with nitrogen gas, essentially as previously described [62], [63], at 25 psi to obtain fragments of 0.8–1.5 kb. Sheared DNA was ethanol precipitated, resuspended in 0.2 ml sterile water, and stored at -20°C until use in microarray experiments.

### Microarray design and printing

The 3,229 70-mer oligonucleotides representing ORFs from *E. faecalis*, including 3093 chromosomal ORFs of strain V583, that were used as probes on the microarrays in this study have been described in detail elsewhere [64]. Approximately 2340 protein coding ORFs (87%) of the OG1RF genome, as annotated in our previously published RAST server annotation [11], were represented on the slides. Oligos were spotted in quadruplicate on GAPS II coated slides (Corning Incorporated, Corning, New York) with a Biorobotics Microgrid II spotter (Digilab, Inc., Holliston, MA) at the University of Minnesota Genomics Center. Spotted arrays were UV-crosslinked 24 hours after printing and were stored in a dessicator. Slides were incubated at 42°C in prehybridization buffer immediately before use, as described in the Corning GAPS II manual.

### Microarray probe preparation and hybridization

Sheared gDNA, which was used as a reference in the microarray experiments [62], was labeled with Cy3-dUTP (GE Healthcare Life Sciences, Piscataway, NJ) using the BioPrime Array CGH Genomic Labeling Module kit (Invitrogen Corp.). Total RNA (9 µl) was reverse transcribed and labeled with Alexa Fluor 647-aha-dUTP using the SuperScript Direct cDNA Labeling System (Invitrogen Corp.). Labeled nucleic acids were purified with the QIAquick PCR purification kit (Qiagen Inc.) and eluted with two 30 µl volumes of 10 mM Tris-Cl, pH 8.5, that were pooled together. Labeled cDNA was mixed with 0.5 µg labeled gDNA, dried down to ∼10 µl in a speedvac, and mixed with ∼40 µl of hybridization probe solution that contained a final concentration of 50% formamide, 5X SSC, 0.1 mg/ml salmon sperm DNA, and 0.1% SDS. The probe was incubated at 95°C for 5 min and cooled at room temperature. Hybridizations were performed at 42°C for 17–19 hours in a MAUI hybridization system (BioMicro Systems, Salt Lake City, UT) using the appropriate MAUI mixers. Slides were then washed twice with 2X SSC, 0.5% SDS, once with 1X SSC, and once with 0.1X SSC before being dried by low-speed centrifugation for 10 min. Slides were scanned on a ScanArray 5000 scanner at a resolution of 10 µm (Perkin Elmer, Waltham, MA). A control experiment was performed with RNA extracted from uninfected chamber aspirate to assess background hybridization from the eukaryotic component of samples. Fluorescence was minimal (data not shown), so the contribution of eukaryotic RNA to the measured signal intensities in experiments containing bacterial RNA was considered negligible.

### Microarray data analysis

The microarray experimental design employed a reference sample design, in which a constant amount of labeled gDNA served as the common reference sample for cDNA samples across all time points studied [62], [65]. This experimental design is advantageous over cDNA/cDNA pair-wise comparison experimental designs for time course studies because it reduces the number of hybridization arrays and amount of RNA per sample required [62]. Scanned images were imported into BlueFuse version 3.6 or earlier (BlueGnome Ltd., Cambridge, UK), where spots were picked using Bayesian statistical modeling to extract the fluorescence intensities. Data were then filtered based upon intensity, Bluefuse confidence, and spot size. Due to a malfunction during slide printing, block 1 spots were uninterpretable and were removed from the analysis at this step. Replicate spots were fused by averaging the intensities of high quality spots. The filtered raw values were exported for further analysis in Expressionist Analyst version 5.3.2 or earlier software (Genedata Inc., Basel, Switzerland). The ratio of the cDNA intensity to the gDNA intensity for each fused spot was calculated and the data from all chips were quantile normalized, as previously described for analyzing gene expression in bacterial systems [62]. Principal components analysis was used to visualize variability among biological replicates while ensuring they clustered together by time point (data not shown). The fold change for each gene was calculated by dividing the cDNA/gDNA ratio obtained at either of the sampled time points (i.e., two or eight hours post-inoculation) by the corresponding cDNA/gDNA ratio of the input inoculum (i.e., zero hours) for each biological replicate. Individual fold changes were then averaged to obtain the final average fold change. The values for down-regulated genes are reported as the negative inverse of the calculated fold-changes, unless otherwise specified. Paired t-tests comparing the cDNA/gDNA ratio for each gene in each time point analysis (i.e., 0 versus 2 hours, 0 versus 8 hours) were performed. Results were filtered for genes showing at least a 2-fold change in expression with a p-value of less than 0.05 and a false discovery rate p-value of less than 0.05. For ease of analysis, data from only those spots known to represent genes in the genome of *E. faecalis* OG1RF, as determined by cross-referencing the gene lists with our previously published RAST genome annotation [11], were included in the final analysis.

### Bioinformatics analysis

Bioinformatics analysis of the gene lists were performed with the Database for Annotation, Visualization and Integrated Discovery (DAVID, v6.7, http://david.abcc.ncifcrf.gov/) [66], [67]. Uniprot Accession numbers were entered to create working gene lists that were analyzed with the KEGG and GOTERM_BP_FAT functions. Results were viewed using DAVID’s Functional Annotation Clustering method. Additional analysis of pathways and gene functions in *E. faecalis* was performed by searching the KEGG Pathway Map and Brite hierarchy functions available at www.genome.jp/kegg/ [68], [69].

### Microarray data accession number

Microarray data from this study has been submitted to the Gene Expression Omnibus (GEO) database [70], accession number GSE22391, to comply with the MIAME standards.

### Quantitative PCR primers, cDNA, and amplification

Oligonucleotide sequences for primers used in qPCR assays are listed in S3 Table. DNase-treated total RNA was reverse-transcribed with random primers using the SuperScript III First-Strand Synthesis System for RT-PCR (Invitrogen Corp.). qPCR was carried out on an iCycler equipped with an iQ5 real-time detection system (Bio-Rad Laboratories, Inc., Hercules, CA) with iQ SYBR Green Supermix (Bio-Rad Laboratories, Inc.). Each reaction was performed in triplicate and the average C_t_ value was used in calculations. DNase-treated RNA was used as a template in control reactions to confirm removal of DNA. EF0886, which was identified in the microarray analysis as being a gene with non-changing expression at the analyzed time points, was used as a reference gene. The fold change for each sample was calculated using the Pfaffl method [71], and the average fold change for each gene assayed was obtained by averaging the fold changes from all biological replicates.

### Blood and serum survival experiments

Human whole blood was obtained from the University of Rochester Medical Center blood bank. Serum was collected from centrifuged whole blood that was coagulated with 20 mM CaCl_2_. Overnight cultures of OG1RF and the (p)ppGpp mutant strains grown in BHI broth were diluted 1∶20 in whole blood or serum and incubated at 37°C. Aliquots collected at 0, 3 (blood only), 6 (serum only), 9, 24, 48, and 72 hours were serially diluted and plated on tryptic soy agar to assess bacterial survival. The Δ*rsh*Δ*relQ* strain caused blood to clot by 24 hours through an as-yet-uncharacterized mechanism (C. Colomer-Winter and J. A. Lemos, unpublished data). Sonication of the clotted blood yielded an increase of one log_10_ CFU/ml in viable counts relative to clotted blood; therefore, quantitative culture results for Δ*rsh*Δ*relQ* strain were adjusted accordingly. Experiments were performed in triplicate and the results were averaged. Results are reported as percent survival with the 0 hour time point set to 100%. Statistical comparisons were calculated with two-way ANOVA followed by Dunnett’s multiple comparisons test in Graphpad Prism. Significance was assessed at α = 0.05.

## Supporting Information

S1 Fig Reverse transcription-qPCR validation of selected differentially expressed genes identified by microarray analysis. *E. faecalis* OG1RF RNA extracted from two and eight hour post-infection subdermal chamber aspirates was reverse transcribed with random hexamers. The resulting cDNAs were used as templates in qPCR experiments. EF0886, which was shown to be stably expressed across time points in the microarray experiments, was used as a reference gene to calculate relative fold change for each gene shown. Data are the mean ± standard deviation of four biological replicates for microarray values and three biological replicates for qPCR values. Down-regulated genes have fold-changes less than one (dotted line). (A) Two hours post-inoculation, (B) Eight hours post-inoculation.
(JPG)

S2 Fig Recovery of OG1RF, Δ*rsh*, Δ*relQ*, and Δ*rsh*Δ*relQ* from subdermal abscesses at (A) early and (B) late time points post-inoculation. Subdermal abscess infections with the four strains were carried out as described in the text and in the legend of Fig. 1. Values and error bars represent the mean ± SEM of n = 2 rabbits. Data from the same rabbits were separated into panels (A) and (B) for clarity.
(TIF)

S1 Table *E. faecalis* OG1RF *in* *vivo* differentially regulated genes identified via microarray analysis at two hours post-infection of subdermal chambers. The fold change values for up- and down-regulated genes are shown in green and red fonts, respectively. Column H lists the corresponding positive and negative fold changes, highlighted in green and red, respectively, from the eight hour microarray.
(XLS)

S2 Table *E. faecalis* OG1RF *in* *vivo* differentially regulated genes identified via microarray analysis at eight hours post-infection of subdermal chambers. The fold change values for up- and down-regulated genes are shown in green and red fonts, respectively. Column H lists the corresponding positive and negative fold changes, highlighted in green and red, respectively, from the two hour microarray. Genes with text in blue font are identical or highly similar to *E. coli* stringent response genes [50]. Genes in boldface text were also differentially expressed in *E. faecalis* in a (p)ppGpp-dependent manner during stringent response activation following 15 or 30 minute treatment with mupirocin [51].
(XLS)

S3 Table List of oligonucleotides.
(DOCX)
