# Supplementary figures and images for: Transcriptome Analysis of Enterococcus faecalis during Mammalian Infection Shows Cells Undergo Adaptation and Exist in a Stringent Response State
Source: PLoS One. 2014 Dec 29;9(12):e115839. doi: 10.1371/journal.pone.0115839 (PMC4278851; doi:10.1371/journal.pone.0115839)

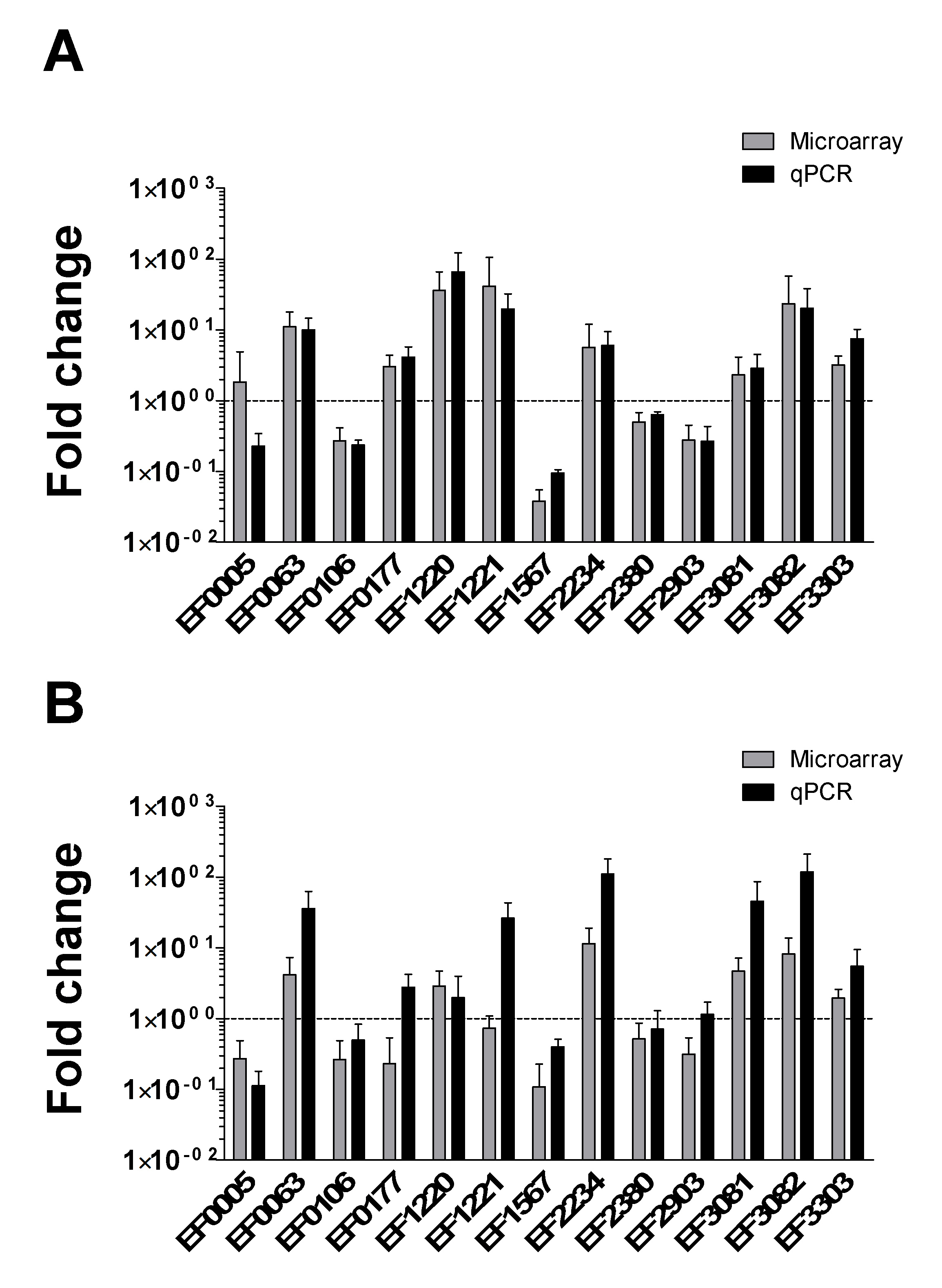

Supplement: S1 Fig — Reverse transcription-qPCR validation of selected differentially expressed genes identified by microarray analysis. E. faecalis OG1RF RNA extracted from two and eight hour post-infection subdermal chamber aspirates was reverse transcribed with random hexamers. The resulting cDNAs were used as templates in qPCR experiments. EF0886, which was shown to be stably expressed across time points in the microarray experiments, was used as a reference gene to calculate relative fold change for each gene shown. Data are the mean ± standard deviation of four biological replicates for microarray values and three biological replicates for qPCR values. Down-regulated genes have fold-changes less than one (dotted line). (A) Two hours post-inoculation, (B) Eight hours post-inoculation. (JPG) [file pone.0115839.s001.jpg]

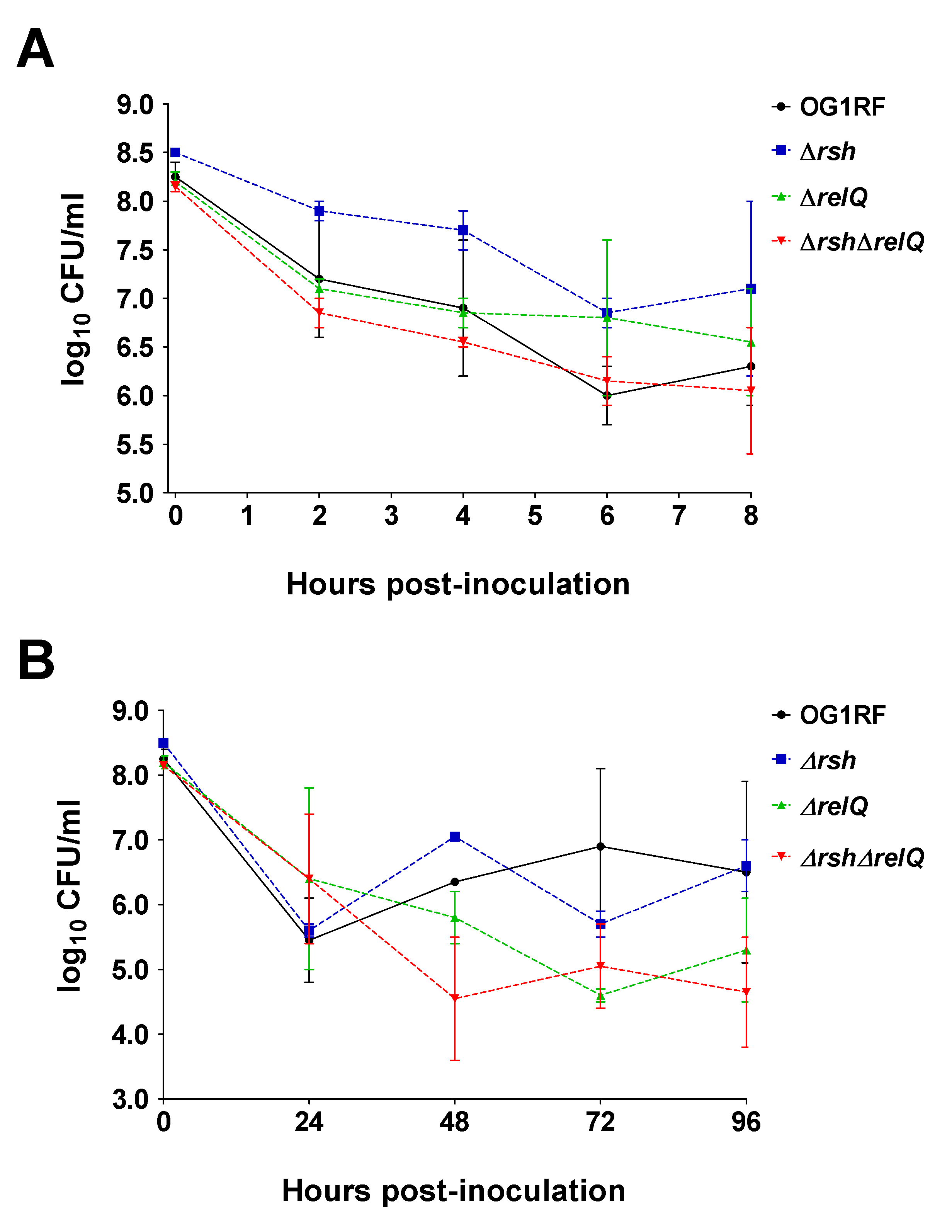

Supplement: S2 Fig — Recovery of OG1RF, Δrsh, ΔrelQ, and ΔrshΔrelQ from subdermal abscesses at (A) early and (B) late time points post-inoculation. Subdermal abscess infections with the four strains were carried out as described in the text and in the legend of Fig. 1. Values and error bars represent the mean ± SEM of n = 2 rabbits. Data from the same rabbits were separated into panels (A) and (B) for clarity. (TIF) [file pone.0115839.s002.tif]
