# Supplementary material for: Transcriptome Analysis of Enterococcus faecalis during Mammalian Infection Shows Cells Undergo Adaptation and Exist in a Stringent Response State
Source: PLoS One. 2014 Dec 29;9(12):e115839. doi: 10.1371/journal.pone.0115839 (PMC4278851; doi:10.1371/journal.pone.0115839)
Supplement: S3 Table — List of oligonucleotides. (DOCX) [file pone.0115839.s005.docx]

| **Oligonucleotide** | **Sequence** | **Source** |
| --- | --- | --- |
| EF0005F (gyrB forward) | caagccaaaacaggtcgcc | **Bourgogne et al. (2007) J Bacteriol 189:6490-6493.** |
| EF0005R (gyrB reverse) | accaacaccgtgcaagcc | Bourgogne et al. (2007) J Bacteriol 189:6490-6493. |
| EF0063F | cggattacgcagatccaagt | This study |
| EF0063R | gctgaagcctcgatcaactc | This study |
| EF0106F | ATCCAGCAGATGAGGCATTC | This study |
| EF0106R | TTGGCTTAGGACTTGGAACG | This study |
| EF0177F | cgtgatcaagatgctgatgg | This study |
| EF0177R | tgtcttctaacgcacggttg | This study |
| 5-EF1220RT | actgtcgctgtccgaacatt | This study |
| 3-EF1220RT | ctcagctggaaaagccaagt | This study |
| EF1221F | gaaaagcgagagcaaaggtg | This study |
| EF1221R | ggtctgcgctattgcctact | This study |
| 5-EF1567RT | gggtgcgggtaaaacaacta | This study |
| 3-EF1576RT | cgattttctggtccgacaac | This study |
| EF2234F | cagcatgtggtggttcaagt | This study |
| EF2234R | ctcaaattctggggtcgtgt | This study |
| EF2380F | ccgttacgctcaactacgaac | This study |
| EF2380R | gcagaagctgggttgaaaga | This study |
| EF2903F | cgcacagctggttcagataa | This study |
| EF2903R | atagccaccagcttcagcat | This study |
| EF3081F | gattgccggcagtagtgaat | This study |
| EF3081R | atcgttcgcgcatcttctac | This study |
| EF3082F | tgtggtagatcgcaccaaag | This study |
| EF3082R | tccaccaccgcttagatacc | This study |
| 5-EF3303RT | ccacgtaatcgttgctgagac | This study |
| 3-EF3303RT | catgaactagggggaagttgg | This study |
| relA-F | caagatttacgggtcattatgg | This study |
| relA-R | gactaatccctaagcgat gtg | This study |
| relQ-F | gacggctattcggcatattcc | This study |
| relQ-R | aagtgcgactacctggtaaatg | This study |

Table S3. List of oligonucleotides.
